# High proportion of unknown HIV exposure status among children aged less than 2 years: An analytical study using the 2015 National AIDS Indicator Survey in Mozambique

**DOI:** 10.1371/journal.pone.0231143

**Published:** 2020-04-07

**Authors:** Maria Grazia Lain, Sergio Chicumbe, Aleny Couto, Esmeralda Karajeanes, Carlo Giaquinto, Paula Vaz

**Affiliations:** 1 Fundação Ariel Glaser contra o SIDA Pediátrico, Maputo, Mozambique; 2 Department for Woman and Child Health, University of Padua, Padua, Italy; 3 Health System Program, Instituto Nacional de Saúde, Maputo, Mozambique; 4 HIV/STI Program, Ministry of Health, Maputo, Mozambique; Johns Hopkins School of Public Health, UNITED STATES

## Abstract

**Background:**

Determination of the human immunodeficiency virus (HIV) exposure status in infants and young children is required to guarantee timely diagnosis and access to appropriate care. HIV prevalence among Mozambican women aged 15–49 years is 15%, and vertical transmission rate is still high. The study investigated HIV exposure in children aged less than 2 years in Mozambique and the factors associated with unknown HIV exposure and with HIV exposure status in this population.

**Methods:**

This was a cross-sectional analytical study using data from the 2015 Survey of Indicators on Immunization, Malaria and HIV/AIDS in Mozambique. A total of 2141 mothers (15–49 years) with children aged less than 2 years were interviewed. The dependent variables were “known HIV exposure status in a child” and “HIV-exposed child,” and the explanatory variables were mother’s social, demographic, economic, and reproductive health characteristics. We used binary and logistic regression, adjusted for complex sampling, to determine the association between variables.

**Results:**

HIV exposure status was unknown in 27% of children (95% CI, 25.1–28.9). Mothers residing in the North (AOR, 4.41; 95% CI, 2.18–8.91), in rural area (AOR, 2.44; 95% CI, 1.33–4.35), with no education (AOR, 2.72; 95% CI, 1.38–5.36), and not having utilized any health services in the last pregnancy (AOR, 1.9; 95% CI, 1.42–2.55) were more likely to have a child with unknown HIV exposure status. Six percent of children were HIV-exposed (95% CI, 5–7). Children were less likely to be HIV-exposed if the head of the household was a male (AOR, 0.26; 95% CI, 0.08–0.86), if the mother was residing in the North (AOR, 0.41; 95% CI, 0.26–0.66) and did not utilize any health services in her last pregnancy (AOR, 0.52; 95% CI, 0.32–0.83).

**Conclusion:**

The high proportion of children with unknown HIV exposure status and the associated socioeconomic factors suggests that HIV retesting of eligible women throughout breastfeeding should be intensified and identifies the urgent need to reach women without prior access to health care using a multisectoral approach.

## Introduction

In 2011, the WHO/UNAIDS Global Plan to eliminate pediatric human immunodeficiency virus (HIV) by 2015 was launched covering all low- and middle-income countries and focusing on 23 priority countries with the highest HIV burden [1]. The plan focused on HIV prevention, treatment, and care interventions to reach all pregnant women living with HIV and their children along the continuum of health care, during pregnancy and throughout breastfeeding. In 2014, despite achieving a 78% antiretroviral therapy (ART) coverage in pregnant women and a 48% decrease in new pediatric infections globally following the adoption of lifelong ART to all pregnant women living with HIV (Option B+), approximately 170,000 newly HIV-infected children were born in priority countries [2]. Approximately 1.2 million women of reproductive age (15–49 years) were living with HIV, and the number of those who newly acquired the virus was still high with an estimated 540,000 new infections in high HIV burden countries, with over 1 million HIV-exposed infants worldwide annually [2].

Mozambique is one of the priority countries included in the global plan. The average HIV prevalence in adults (15–49 years) is 13.2% in this country, ranging from 5.2% in Tete (Central region) to 24.4% in Gaza Province in the Southern region [3]. It is estimated that 2.1 million people are currently living with HIV; among these, 59% are aware of their HIV status, and 54% are on ART [4]. Since the launch of Option B+ in 2013, a remarkable progress has been observed, and encouraging results have been achieved. By the end of 2015, 95% of women attending the antenatal care clinics (ANCs) were tested for HIV, and 91% received ART. The vertical transmission rate decreased to 9%, and the incidence of new pediatric infections decreased by nearly 70% since 2009 [2,5]. However, the identification of all HIV-exposed infants and vertically infected children was not being possible as early as desirable, considering that only 43% of infants had access to virologic testing within the first 2 months of life [2,5].

Along the prevention of mother-to-child transmission (PMTCT) cascade, several ongoing challenges preclude reaching all women living with HIV and their HIV-exposed infants. In Mozambique, similar with the other priority countries in the African region [6], HIV testing is offered as per the WHO recommendations [7] at the ANCs and other mother and child health (MCH) services such as maternity wards, postpartum care clinics, and family planning clinics. However, systematic data collection on HIV retesting during the pregnancy-breastfeeding period in women with a previous negative test is not being performed. A 2011 study on breastfeeding women in Mozambique showed an HIV incidence rate as high as 4.9% up to 18 months after a negative HIV test at delivery [8]. Other similar studies in neighboring countries also reported high incidence of seroconversion of women during pregnancy or postpartum period following an initial negative test [9–12].

To eliminate vertical transmission, it is imperative that all women of childbearing age, who are pregnant or breastfeeding, have continued access to HIV testing, are aware of their HIV status and consequently the HIV exposure status of their children. Certainly, awareness regarding HIV exposure in infants is a prerequisite for the success of the Early Infant Diagnosis Program, early ART initiation in infected infants, and eventually access to HIV cure interventions.

The identification of factors associated with the status of “unknown HIV exposure” and of “HIV exposure” in infants and young children is key to identify the gaps in the provision of health services to mother-child pairs, to guide the development of focused interventions to eliminate vertical transmission, and to end the AIDS epidemic by 2030.

To the best of our knowledge, there have been no population-based studies describing these factors in Mozambique or in other countries. Therefore, this study aimed to evaluate the characteristics associated with unknown HIV exposure status and HIV-exposed status in infants and children aged less than 2 years in Mozambique.

## Methods

### Study design

This was a cross-sectional analytical study that used data collected during the 2015 Survey of Indicators on Immunization, Malaria and HIV/AIDS in Mozambique (IMASIDA) [3]. The primary aim of the survey was to estimate the following: HIV prevalence in the adult population, malaria prevalence, and immunization coverage in children aged less than 5 years in Mozambique. Besides the standard “household questionnaire,” the survey included a “woman and child health questionnaire” for women of childbearing age, focusing on malaria, immunization, maternal and child health, and HIV. The survey used a standardized questionnaire designed by the Demographic and Health Surveys (DHS) program, which is available for free download [13]. The questionnaire captured HIV indicators in women and children, which included HIV testing of women at the ANCs, at delivery, and during the postnatal period, the HIV test result at each of the previous contacts with the health services, and HIV testing offered to the child. Standard questions regarding mother and child health, including utilization of healthcare services, medicines taken, and infant’s feeding and immunization practices, were also asked. The survey was conducted between June 2015 and September 2015 using a systematic, multistage, stratified, and proportionate-to-size sampling [3]. The overall survey was designed to obtain the HIV prevalence estimates with 95% confidence intervals (CIs), an error margin of 5%, and a power of 80% and to ensure the representation of rural and urban areas and provincial levels. In the survey, a total of 8204 women were eligible for the interview, 7749 women consented for the same and 6946 women of childbearing age (15–49 years) were interviewed using the “woman and child health questionnaire” [3]. For the purpose of our analysis, we restricted the database to 2141 women aged between 15–49 years with at least one child aged less than 2 years on the day of the interview. Weights were computed by the DHS program [14] and provided as variable in the accessed database, allowing to adjust our analysis for unequal selection probabilities. The DHS program, funded by the US Agency for International Development (USAID), routinely conducts nationally representative surveys, including AIDS Indicator Survey, worldwide and generally in low- and middle-income countries.

### Dependent variables

An HIV-exposed infant/child is an infant/child born to an HIV-positive mother or an infant who has been breastfed or is currently breastfed by an HIV-positive mother and whose definitive diagnosis has not yet been established up to the day of the interview [15].

The analysis considered the following two dependent variables: (1) “known HIV exposure status in a child,” coded as “1” in cases where the “child is known to have been HIV-exposed” or is “known to not have been HIV-exposed” and “0” if HIV exposure could not be ruled out, in cases of “unknown HIV exposure status in a child” and (2) “HIV-exposed status in a child,” coded as “1” if the child is “exposed to HIV,” coded as “0” if a child is “not exposed to HIV,” and coded as “8” in cases of “unknown HIV exposure status.” The variable “HIV-exposed status in a child” was computed by the DHS program and provided in the accessed database.

### Independent variables

Studies evaluating the determinants of access to and utilization of HIV prevention and care services constituted the basis for defining the key independent variables [16–20]. Besides the woman’s demographic characteristic, educational attainment, employment, religion, area and region of residence, socioeconomic status, and amenities of the households, the independent variables included the variables of health service utilization. Health service utilization was defined as the “use of antenatal care services, maternity, and postnatal care services.” Wealth index was provided as computed by the DHS program [14], which used a factor analysis for household amenities such as television, radio, refrigerator, bicycle, motorcycle, computer, water supply, sanitation, and cooking fuel [14]. Using transformations, we computed the following nominal independent variables: “media utilization” was defined as owing a television (TV) or a radio and watching TV or listening to the radio at least once a week, “mean of transport” was defined as owing a bicycle, or a motor bike or a car, and “immunization status of the child” according to the age-adjusted up-to-date vaccination for diphtheria-tetanus-pertussis (DPT) and measles. We used DPT1 (DPT dose 1), DPT2 (DPT dose 2), DPT3 (DPT dose 3), and Measles1 as the representative vaccines (“tracers”) considering that the other vaccines (rotavirus, HepB, Hib, polio, pneumococcal conjugate vaccine 10) are simultaneously administered with the tracer vaccines. Immunization was defined as up-to-date if the child had DPT1 by 2 months; DPT1 and DPT2 by 3 months; DPT1, DPT2, and DPT3 by 4 months, and DPT1, DPT2, DPT3, and measles by 24 months. If any of these age-adjusted vaccines were not reported, the immunization status of the child was considered “not up-to-date.” “Mother’s participation in family decisions” was obtained based on the answers to the questions on who decides on (1) household financial management, (2) healthcare utilization, (3) household purchases and expenses, and (4) visits to and from relatives. Participating in the decision-making was coded “1” if the mother decided alone or with other members on all of the described aspects and “0” if she reported no participation in one or more of the described aspects.

### Statistical methods

First, the results of the descriptive analysis were expressed as absolute and relative frequencies with respective 95% CIs. Second, analyses were performed to identify the factors associated with “known HIV exposure status in a child” and “HIV-exposed status in a child” against the explanatory variables by bivariate association assessed by *p*, odds ratio (OR), and 95% CI. Third, binary logistic regression was used to estimate the adjusted OR (AOR) and 95% CI of being a child with unknown HIV exposure status and of being an HIV-exposed child against the socioeconomic, demographic, and health service utilization covariates. The association analysis was weighted and adjusted for complex sampling using the statistical package International Business Machines Corporation Statistical Package for the Social Sciences Statistics for Windows version 24.0 [21].

### Ethical considerations

The 2015 IMASIDA Survey was approved by the Mozambican National Bioethics Committee (IRB00002657, reference 262/CNBS/2014) and the Ministry of Health. The participation was voluntary through a signed informed consent administered by a trained interviewer. For illiterate participants, the informed consent process was witnessed by a literate person chosen by the participant. For participants aged 15–17 years, signed informed consent was obtained from the legal guardian and assent from the participant.

For this secondary data analysis, authorization for the use of database has been granted by the DHS program [22], the database was de-identified, and no informed consent was required from the participants. The database is publicly available through an application process to the DHS program site.

## Results

### Socioeconomic characteristics of the study participants

The social and demographic characteristics of our study sample are described in Table 1. Among the 2141 interviewed women, a total of 1769 (83%; 95% CI, 81.5–84.8) were breastfeeding at the time of interview, and 66 (3%; 95% CI, 2.4–3.8) were pregnant. Approximately 41% of women had four or more children, 68% were aged less than 30 years, and 24.8% (95% CI, 23–26.7) had no education. Twenty-nine percent (95% CI, 27.4–31.3) were from the Northern region and 41.7% (95% CI, 39.6–43.8) from the Central region. Majority of them (63.8%; 95% CI, 61.8–65.9) were living in a rural area, and 68.9% (95% CI, 67–71) did not have radio or television at home. The head of the household was male in 63% (95% CI, 60.9–65.1) of the households, and 10.3% (95% CI, 9–11.6) of women participated in the decision-making process in the family.

**Table 1:** Characteristics of interviewed mothers- Mozambique 2015 (N = 2141).

		All (n = 2141)			
		n	%	95% CI	
**Sex of household head**	Male	1348	63.0	60.9	65.1
** **	Female	793	37.0	35.0	39.1
**Mother's Age (years)**	15–19	406	19.0	17.3	20.6
** **	20–24	607	28.4	26.4	30.3
** **	25–29	448	20.9	19.2	22.7
** **	30–34	313	14.6	13.1	16.1
** **	35+	367	17.1	15.5	18.8
**Participation in family decisions**	No	1920	89.7	88.4	91.0
** **	Yes	221	10.3	9.0	11.6
**Mother's educational level**	No education	531	24.8	23.0	26.7
** **	Primary	1135	53.0	50.9	55.2
** **	Secondary	475	22.2	20.4	24.0
**Mean of transport**	No	1190	55.6	53.5	57.7
** **	Yes	951	44.4	42.3	46.6
**Source of water at home**	No piped water	1325	61.9	59.8	64.0
** **	With piped water	816	38.1	36.1	40.2
**Toilet**	Not improved	1863	87.0	85.6	88.5
** **	Improved	278	13.0	11.6	14.4
**Cooking fuel**	Improved	62	2.9	2.2	3.6
** **	Coal or Wood	2053	95.9	95.0	96.8
** **	Not applicable	26	1.2	0.8	1.7
**Media utilization**	No	1476	68.9	67.0	71.0
** **	Yes	665	31.1	29.1	33.0
**Region of residence**	North	628	29.3	27.4	31.3
** **	Center	892	41.7	39.6	43.8
** **	South	621	29.0	27.1	31.0
**Place of residence**	Urban	775	36.2	34.2	38.3
** **	Rural	1366	63.8	61.8	65.9
**Nr of live children**	1	526	24.6	22.7	26.4
** **	2	397	18.5	16.9	20.2
** **	3	338	15.8	14.2	17.3
** **	4	261	12.2	10.8	13.6
** **	5+	619	28.9	27.0	30.9
ANC* **consultation (n)**	None	134	6.3	5.2	7.3
** **	1–3	652	30.5	28.5	32.4
** **	4+	1355	63.3	61.2	65.4
**Mother HIV test at ANC or Delivery**	Not done	346	16.2	14.6	17.7
** **	done	1650	77.1	75.3	78.9
** **	Unknown	145	6.8	5.7	7.8
**Institutional Delivery**	No	550	25.7	23.8	27.6
** **	Yes	1591	74.3	72.5	76.2
**Place of delivery**	Respondent's home	472	22.1	20.3	23.9
** **	Other home	43	2.0	1.4	2.6
** **	Hospital	601	28.1	26.2	30.1
** **	Health center	722	33.8	31.8	35.8
** **	Health post	261	12.2	10.8	13.6
** **	Clinic	7	.3	0.1	0.6
** **	Other private	6	.3	0.1	0.5
** **	Other	24	1.1	0.7	1.6
** **	Missing	5			
**Current age of last child**	0	1033	48.2	46.1	50.4
** **	1	1014	47.4	45.2	49.5
** **	2	94	4.4	3.5	5.3
**Breastfeeding duration of last child**	≤ 6 months	62	2.9	2.2	3.6
** **	≤ 12 months	125	5.9	4.9	6.9
** **	≤ 18 months	148	7.0	5.9	8.0
** **	Currently breastfeeding	1769	83.1	81.5	84.8
** **	Never breastfed	24	1.1	0.7	1.6
** **	Missing	13			
**Infant's postnatal visit within 2 months of life**	No	421	19.7	18.0	21.4
** **	Yes	1648	77.2	75.4	79.0
** **	Don't know	66	3.1	2.4	3.8
** **	Missing	6			
**Child Immunization up-to-date**	No	468	21.9	20.1	23.6
** **	Yes	1673	78.1	76.4	80.0
**Currently pregnant**	No or unsure	2075	96.9	96.2	97.7
** **	Yes	66	3.1	2.4	3.8
Utilization of Health Services (ANC, Maternity and PNC**)	No	1176	54.9	52.8	57.1
** **	Yes	965	45.1	43.0	47.2
**Self-reported HIV status of previous HIV test**	Positive	141	6.6	5.5	7.7
** **	Negative	1520	71	69.1	73.0
** **	Indeterminate	10	0.5	0.2	0.8
** **	Refused to answer	7	0.3	0.1	0.5
** **	Did not receive result	81	3.8	3.0	4.6
** **	Never tested	382	17.8	16.2	19.4
**Child exposed to HIV**	Not exposed	1435	67.0	65.0	69.1
** **	Exposed	129	6.0	5.0	7.0
** **	Unknown	577	27.0	25.1	28.9
	Total	2141	100.0		

*ANC: Antenatal care

**PNC: Postnatal care

### Health service utilization

Regarding last pregnancy, 93.7% of women reported to have attended the ANC clinics, 63.3% (95% CI, 61.2–65.4) completed four or more ANC visits, 74.3% (95% CI, 72.5–76.2) gave birth in a health facility, and 77.2% (95% CI, 75.4–79) had a postnatal consultation within the infant’s second month of life. Immunization in 78% (95% CI, 76.4–80) of children was up-to-date. Seventy-seven percent (95% CI, 75–78.9) of women had an HIV test at the ANC or at delivery, 6.8% (95% CI, 5.7–7.8) were not aware if the test was performed or not, 71% (95% CI, 69.1–73) reported a negative result, 6.6% (95% CI, 5.5–7.7) reported a positive result, 17.8% (95% CI, 16.2–19.4) reported to never have been tested in the past, and 3.8% (95% CI, 3–4.6) reported to not have received the test result. The proportion of mothers with child of unknown HIV exposure status was 27% (95% CI, 25–28.9); in children with known HIV exposure status, 6% (95% CI, 5–7) were HIV-exposed.

### Factors associated with unknown HIV exposure status in a child

In bivariate analyisis, factors associated with “unknown HIV exposure status” in a child were as follows (Table 2): household head being a male (OR, 1.31; 95% CI, 1.032–1.68), no education or primary educational level of the mother (OR, 7.98; 95% CI, 4.67–13.64 and OR, 4.47; 95% CI, 2.81–7.12, respectively), residence in the Northern region (OR, 8.84; 95% CI, 5.38–13.13) or in the Central region of the country (OR, 6.95 95%; CI 4.57–10.58), age of the youngest child between 1 and 2 years (OR, 2.04; 95% CI, 1.13–3.81), duration of breastfeeding between 6 and 12 months (OR, 5.33; 95% CI, 1.31–21.63), child being breastfed currently (OR, 4.67; 95% CI, 1.23–17.7), belonging to the poorer categories of wealth index (OR, 11.23; 95% CI, 6.3–19.9), mother with 1–3 visits to the ANC clinic (OR, 2.16; 95% CI, 1.60–2.91), mother not having utilized any health services during and after her last pregnancy (ANC visit, maternity, postnatal consultation) (OR, 2.64; 95% CI, 1.85–3.76), and child’s immunization not being up-to-date (OR, 2.16; 95% CI, 1.59–2.93). Children were less likely to have an “unknown HIV exposure status” when the mother was aged between 30 and 34 years (OR, 0.56; 95% CI, 0.37–0.84), was residing in an urban area (OR, 0.20; 95% CI, 0.14–0.29), and had four children (OR, 0.59; 95% CI, 0.39–0.90).

**Table 2:** Factors associated with unknown HIV exposure status in a child—Mozambique 2015.

Unknown HIV exposure status in child		Bivariate analysis				Multivariate analysis			
		*p value*	OR	95% CI		*P value*	Adjusted OR	95% CI	
				Lower	Upper			Lower	Upper
**Sex of household head**	Male vs. Female	.027	1.317	1.032	1.680	.104	1.285	.950	1.738
Participation in family decision*	No vs. Yes	.052	1.547	.996	2.403	-	-	-	-
**Mother's age (years)**	15–19 vs. 35+	.155	.773	.543	1.102	.318	.731	.394	1.354
	20–24 vs. 35+	.097	.744	.524	1.056	.827	.944	.563	1.582
	25–29 vs. 35+	.076	.724	.507	1.035	.552	.873	.558	1.368
	30–34 vs. 35+	.005	.560	.372	.841	.212	.736	.454	1.192
**Highest educational level**	No education vs. Secondary	.000	7.986	4.674	13.643	.004	2.726	1.386	5.361
	Primary vs. Secondary	.000	4.475	2.812	7.120	.031	1.826	1.057	3.153
**Wealth index**	Poorest vs. Richest	.000	11.195	6.380	19.643	.391	1.464	.611	3.505
	Poorer vs. Richest	.000	11.234	6.313	19.993	.117	1.985	.842	4.678
	Middle vs. Richest	.000	8.556	4.703	15.564	.168	1.787	.781	4.087
	Richer vs. Richest	.018	2.178	1.146	4.138	.876	1.059	.511	2.198
**Place of residence**	Urban vs. Rural	.000	.205	.144	.292	.004	.419	.233	.753
**Region of residence**	North vs. South	.000	8.408	5.381	13.138	.000	4.411	2.181	8.919
	Center vs. South	.000	6.959	4.573	10.588	.000	3.015	1.861	4.886
**Number of live children**	1 vs. 5+	.587	.916	.668	1.257	.044	1.810	1.016	3.225
	2 vs. 5+	.016	.641	.447	.921	.907	.968	.559	1.676
	3 vs. 5+	.437	.838	.535	1.311	.302	1.254	.815	1.928
	4 vs. 5+	.016	.599	.395	.909	.047	.606	.370	.993
**Current age of last child (yr)**	0 vs. 2	.040	1.859	1.029	3.358	.086	1.926	.911	4.075
	1 vs. 2	.017	2.084	1.139	3.811	.039	2.107	1.039	4.276
**Breastfeeding duration of last child**	≤ 6 months vs. Never	.090	3.633	.817	16.157	.067	3.633	.911	14.488
	≤ 12 months vs. Never	.019	5.333	1.315	21.630	.016	4.486	1.321	15.234
	≤ 18 months vs. Never	.147	2.873	.688	12.001	.239	2.286	.576	9.074
	Still breastfeeding vs. Never	.024	4.671	1.231	17.718	.160	2.296	.718	7.341
**Utilization of health services (ANC, Maternity, PNC)**	No vs. Yes	.000	2.640	1.852	3.763	.000	1.905	1.422	2.552
**Child Immunization up-to-date**	No vs. Yes	.000	2.162	1.595	2.930	.161	1.246	.916	1.695

*Variable not analyzed in the Multivariate analysis because its association was not significative in the bivariate analysis.

In multivariate analysis, factors that persisted to be associated with “unknown HIV exposure status” in a child were as follows: residence in the Northern region (AOR, 4.41; 95% CI, 2.18–8.91), residence in the Central region (AOR, 3.01; 95% CI, 1.86–4.88), mother with no education (AOR, 2.72; 95% CI, 1.38–5.36) or with primary educational level (AOR, 1.82; 95% CI, 1.05–3.15), age of the youngest child between 1 and 2 years (AOR, 2.10; 95% CI, 1.03–4.27), duration of breastfeeding between 6 and 12 months (AOR, 4.48; 95% CI, 1.32–15.23), and mother not having utilized any health services during and after her last pregnancy (AOR, 1.9; 95% CI, 1.42–2.55). Children were less likely to have an “unknown HIV exposure status” if the mother was residing in an urban area (AOR, 0.41; 95% CI, 0.23–0.75) and if she had four children (AOR, 0.60; 95% CI, 0.37–0.99).

### Factors associated with an HIV-exposed child

In bivariate analysis (Table 3), the factor associated with being an “HIV-exposed child” was “mother residing in an urban area” (OR, 1.55; 95% CI, 1.00–2.40). Children were less likely to be HIV-exposed if the household head was a male (OR, 0.47; 95% CI, 0.31–0.70), the mother’s age was between 15 and 19 years (OR, 0.27; 95% CI, 0.12–0.64) and between 20 and 24 years (OR, 0.33; 95% CI, 0.16–0.68), the mother was residing in the Central region (OR, 0.30; 95% CI, 0.18–0.50) and in the Northern region (OR, 0.13; 95% CI, 0.05–0.31) of the country; the mother belonged to the poorer wealth category (OR, 0.13; 95% CI, 0.04–0.40), the mother had one child (OR, 0.39; 95% CI, 0.21–0.71), the mother was currently breastfeeding (OR, 0.20; 95% CI, 0.06–0.58), and if she did not utilize any health services during and after her last pregnancy (OR, 0.43; 95% CI, 0.26–0.71).

**Table 3:** Factors associated with the condition ‘HIV-exposed child’- Mozambique 2015.

HIV-exposed child		Bivariate analysis				Multivariate analysis			
		*p value*	OR	95% CI		*P value*	Adjusted OR	95% CI	
				Lower	Upper			Lower	Upper
**Sex of household head**	Male vs. Female	.000	.472	.315	.707	.000	.418	.261	.667
**Mother's age (years)**	15–19 vs. 35+	.003	.277	.120	.642	.208	.393	.091	1.687
	20–24 vs. 35+	.003	.334	.163	.684	.055	.416	.170	1.018
	25–29 vs. 35+	.259	.666	.328	1.352	.412	.720	.327	1.584
	30–34 vs. 35+	.848	1.063	.567	1.995	.655	1.169	.587	2.327
**Number of live children**	1 vs. 5+	.002	.393	.216	.715	.316	.553	.173	1.764
	2 vs. 5+	.069	.532	.270	1.050	.368	.677	.289	1.585
	3 vs. 5+	.697	1.120	.633	1.982	.612	1.205	.584	2.484
	4 vs. 5+	.757	1.099	.601	2.011	.247	1.492	.756	2.943
Highest educational level*	No education vs. Secondary	.055	.518	.265	1.015	-	-	-	-
	Primary vs. Secondary	.869	1.045	.621	1.759	-	-	-	-
Participation in family decision*	No vs. Yes	.970	1.013	.527	1.945	-	-	-	-
**Wealth index**	Poorest vs. Richest	.001	.163	.055	.481	.161	.357	.084	1.512
	Poorer vs. Richest	.000	.134	.044	.406	.056	.296	.085	1.032
	Middle vs. Richest	.533	.820	.438	1.534	.116	1.870	.856	4.083
	Richer vs. Richest	.273	.754	.454	1.252	.814	.932	.516	1.682
**Place of residence**	Urban vs. Rural	.049	1.553	1.002	2.405	.750	.910	.509	1.628
**Region of residence**	North vs. South	.000	.135	.058	.314	.028	.264	.080	.867
	Center vs. South	.000	.304	.184	.503	.006	.430	.235	.787
**Current age of last child (yr)**	0 vs. 2	.562	1.320	.516	3.377	-	-	-	-
	1 vs. 2	.964	.978	.370	2.586	-	-	-	-
**Breastfeeding duration of last child**	≤ 6 months vs. Never	.191	2.352	.651	8.492	.105	3.459	.771	15.515
	≤ 12 months vs. Never	.195	.453	.136	1.504	.630	.698	.161	3.027
	≤ 18 months vs. Never	.004	.081	.015	.436	.009	.070	.010	.512
	Still breastfeeding vs. Never	.004	.200	.068	.587	.101	.342	.095	1.234
**Utilization Health Service (ANC, Maternity, PNC)**	No vs. Yes	.001	.436	.265	.718	.007	.520	.323	.836
Child Immunization up-to-date*	No vs. Yes	.188	.648	.340	1.237	-	-	-	-
Currently pregnant*	No or unsure vs. Yes	.632	1.343	.400	4.516	-	-	-	-

*Variable not analyzed in the Multivariate analysis because it was not significative in the bivariate

In multivariate analysis, children were less likely to “be HIV-exposed” if the head of the household was a male (AOR, 0.41; 95% CI, 0.26–0.66), if the mother was residing in the Northern region (AOR, 0.26; 95% CI, 0.08–0.86) and in the Central region (AOR, 0.43; 95% CI, 0.23–0.78), if the duration of breastfeeding was between 12 and 18 months (AOR, 0.070; 95% CI, 0.01–0.51), and if the mother did not utilize any health services in her last pregnancy (OR, 0.52; 95% CI, 0.32–0.83).

## Discussion

To the best of our knowledge, this is the first population-based study describing the factors associated with the status of unknown HIV exposure among children aged less than 2 years and the factors associated with the status of child being HIV-exposed in a context with a high HIV vertical transmission rate. We found approximately a third of children with an unknown HIV exposure status, and considering that 83% of interviewed women were breastfeeding, this is alarming. We also found that 18% of interviewed mothers reported never having been tested for HIV, and approximately 4% reported that they did not receive the result of the test performed in the past. This is in contrast with the 95% HIV testing coverage among women presenting at first ANC reported by the MOH in 2015 [5].

In Mozambique, offering an HIV test to a pregnant woman at the first ANC visit, and retesting at delivery and every 3 months up to 9 months after the baby is born in case of a negative test result are part of the MCH routine care guidelines [7,23].

However, from our study, it is evident that a significant proportion of women who needed to be tested for HIV along the PMTCT pathway were missed: 6% did not attend any ANC visit during their last pregnancy, 26% delivered outside a healthcare facility, and 20% did not have a postnatal visit within the infant’s second month of life.

Considering the high (over 4%) HIV incidence rate occurring during pregnancy and breastfeeding in previously HIV-uninfected women, reported in Mozambique, our findings are alarming [8,24]. Similar incidence rates, ranging from 4.4 to 16.8%, have been reported in other neighboring countries such as Kenya, South Africa, and Zimbabwe [9–12,25–27]. Pregnancy and postpartum are periods of persistent risk of HIV acquisition for a woman [26], and in this period, they have higher risks of vertical transmission than do women with chronic HIV infection [28].

HIV retesting guidelines have been developed by the WHO [7]; however, their uptake is not optimal. A recent paper reviewed and characterized the African countries’ guidelines on the timing and frequency of maternal retesting. This study reported that in Kenya, South Africa, Tanzania, Uganda, Zambia, and Zimbabwe, regular retesting was part of the norm, but in other countries with a high rate of mother-to-child transmission, they were not consolidated [6].

Poor retesting of eligible women highlights the need to strengthen this practice among healthcare staff and counselors throughout the HIV exposure period for the child. Awareness campaigns on the importance of HIV testing targeting women of child-bearing age, pregnant and breastfeeding need to be spread in the community.

We found that mothers of children with known HIV exposure status had 30 more percentage points of institutional deliveries and had 46 more percentage points of MCH service utilization compared to mothers of infants with unknown HIV exposure status (S1 Table). Another interesting finding is that women with children breastfed up to 12 months had a higher chance of not knowing the infant’s HIV status compared to those who never breastfed [29]. It is likely that, despite the careful attention being provided to mothers during pregnancy and up to delivery, systematic follow-up by healthcare staff and HIV counseling and testing practice during prolonged breastfeeding are relatively neglected.

The vaccination appointment is another opportunity to screen mothers and infants for HIV. A study in Tanzania showed that the integration of HIV testing in immunization clinics can increase the identification of HIV-infected mothers and HIV-exposed infants in the first 2 months after birth [30]. National figures show an immunization coverage at 4 months of 82% and a measles coverage of 83% at 9 months of age [3]; however, in our study, 22% of children had no up-to-date vaccination schedule, a condition more common in infants with unknown HIV exposure status compared to those with known HIV exposure status (S1 Table).

Integration and synergism of existing services such as vaccination and HIV care at the MCH clinic may result in positive outcomes, considering that there is some evidence in Sub-Saharan Africa that the up-take of childhood vaccination is lower in HIV-exposed infants than in HIV-unexposed infants [31]. Nevertheless, the utilization of the immunization platform to improve HIV screening needs to be carefully planned, providing due importance to acceptance by the mothers and healthcare staff [32]. In addition, an efficient and clear patient’s flow within the healthcare facility is required, as reported by mothers in Beira, Central Mozambique, to improve health facility navigation and consequently to increase mother-child retention in care [33].

Other factors in the multivariate analysis associated with “unknown HIV exposure status” in child were residence in Northern and Central regions, where HIV prevalence is lower. A woman living in the Northern and the Central regions had 3–4 times more chances of having a child with unknown HIV status compared to a woman residing in the Southern region. The Northern region of Mozambique, which includes the provinces of Cabo Delgado, Nampula, and Niassa, is poorer and less developed, with the majority of people living in rural areas, a condition that, according to our results, is also associated with a twofold increased chance of having a child with unknown HIV exposure status. Socioeconomic factors, such as poverty and underdevelopment, are possibly associated with “unknown HIV status exposure” in a child because they imply conditions that limit access to healthcare facilities, such as remote residential location, poor road conditions, limited transport availability, or insufficient finances[33]. Similar factors, such as poverty, low literacy, low socioeconomic status, and residence in rural setting, were associated with underutilization of ANC services and low access to institutional delivery in other Sub-Saharan African countries such as Uganda [34], Kenya, [35] and Nigeria [17]. Food insecurity and the need to attend to other household responsibilities have been reported in Mozambique as factors limiting the regular follow-up of women at MCH services [33].

Access to education is another factor directly related to poverty and to living in a rural area, and in general, women have less opportunities to go to school than men. In Mozambique, it has been reported that 35% of women with no education reside in rural areas versus 17% who are residing in urban areas [3]. It is also understood that the opportunity to go to school increases with the increase family wealth index [20]. In our study, majority of women were poor, and those with no education were three times more likely to not know the HIV status of their children compared to those with secondary education. Literacy enables awareness regarding the available services provided by the ANCs and postnatal care services and empowers a woman to seek care for herself and her children and to use the health services as described in Nigeria [17], Kenya [35], and Ghana [20], where no education or primary education is reported to be a factor associated with the underutilization of the ANCs.

Access to media is also limited by poverty, and lack of information can contribute to poor knowledge about HIV prevention and care. In our sample, 69% of interviewed women reported not having television or radio at home, which has been described to be more frequent in Cabo Delgado and Nampula provinces (80%) [3].

Several studies described the association between knowledge of health services and HIV prevention practice, access, and retention into PMTCT care in neighboring countries such as South Africa, Malawi, Tanzania, Uganda, Kenya, and Nigeria [17,36,37]. In Mozambique, only 30% of women and 51% of men aged 15–24 years reported comprehensive knowledge about HIV prevention practices [38]. Despite the initiative of the Ministry of Health and the Institute of Social Communication to produce and broadcast television videos and radio programs in local languages, and to promote communication campaigns with itinerant theatre groups to disseminate information on HIV prevention and early testing, coverage is limited, and only few people are being reached in reality. Another strategy adopted in Mozambique is to telecast videos on HIV prevention and treatment awareness in the waiting area of healthcare facilities, but while we are targeting people attending the clinics, we are not being able to reach people who reside in remote rural areas and do not have access to healthcare facilities.

The study shows that 6% of children aged less than 2 years were HIV-exposed. Considering that the HIV prevalence among women of child-bearing age is 15.4% [3], this result is lower than expected, and in part, it may be due to the fact that 27% of children in our study population had an unknown HIV exposure status.

We found that children were less likely to be HIV-exposed when the duration of breastfeeding was between 12 and 18 months compared to those who never breastfed. In Mozambique, according to the WHO guidelines, breastfeeding is recommended up to 12 months of age in infants with mothers living with HIV [39]. In our context, a mother who is breastfeeding for longer than 12 months is more likely to be HIV negative, with her infant being HIV-unexposed; in fact, mothers who do not breastfeed are usually those who are sick or have any other health contraindications, although no empirical reports have been published.

Having a male as the head of the family was associated with the “unknown HIV exposure status” in a child and also with lower chances of being an HIV-exposed child. The reason is unclear; however we believe that it is associated with cultural behaviors and gender dynamics [36,40]. Families in Mozambique are headed predominantly by males who decide on all family matters; in our study population, only 10% of women participated in the household decision-making process, which may have led to the underutilization of health services. Decisions regarding pregnancy and infant care are often taken by someone else other than the mother, such as an elder female family member, grandmother, or husband’s family members, and it has been shown to affect HIV care adherence and retention among women in Kenya and Malawi [36,41,42].

In a family where a woman is HIV infected, it is common that the partner rejects her and leaves the family [43–45]. In Mozambique and elsewhere, it has been described that fear of stigma, discrimination, and losing their home are barriers for women to get tested and to access health care [24,33,46,47]. De Schacht et al. found that 20% of women were not aware if the husband was HIV tested, indicating that HIV infection is not a topic routinely discussed at home [24]. Other studies in Lesotho, Northern Mozambique and South Africa, reported men’s fear of testing for HIV, lack of education and information about HIV/AIDS and fear of self-disclosure of their HIV status [46,48–50].

The role of men and their contribution to the success of PMTCT requires further investigation considering that male engagement in gender equity and sexual health interventions is crucial to address family health needs, reduce HIV burden, and in our case, eliminate pediatric HIV infection [51].

The analysis has a few limitations. The survey was powered to determine the HIV prevalence in the adult population, and not for the primary purpose of our analysis. The logistic regression did not control for clustering of children at the mother’s level; however, possible clustering is considered negligible as the number of women with more than one child aged less than 2 years was only 3.2% (n = 68). Additionally, as we restricted the sample to women with at least one live child aged less than 2 years, the sample size was reduced, which may be considered as a possible source of selection bias. However, the sample is still large, and the restriction allowed to gain control on the crucial recall bias and to focus the study on the PMTCT target population for the entire 24-month follow-up period. Nondisclosure of serostatus during the survey may have underestimated the number of HIV-exposed children in favor of the overestimation of unknown HIV exposure status. Even if that is the case, results are still valid since the outcomes balance each other in terms of bias direction and share similar, consistent, and programmatically relevant associated factors. The IMASIDA 2015 questionnaire did not explore stigma and discrimination experienced within the household, the health facility or the community as a possible factor contributing to the lack of uptake for HIV testing, and we could not evaluate this potential cause of unknown HIV serostatus.

## Conclusions

The first step to prevent vertical HIV transmission is to determine the mother’s HIV status. Our study points at the persistent missed opportunities to eliminate pediatric HIV in Mozambique as shown by one-third of children having unknown HIV exposure status while their mothers utilized healthcare services, with the majority of them breastfeeding.

Socioeconomic factors such as education, access to information, and poverty are associated with unknown HIV exposure and HIV exposure status in children. Results indicate that a continuum of care to women who have access to healthcare services should be properly provided, and that women who have no access to healthcare services should be reached and engaged into MCH care.

The implementation of HIV testing national guidelines must be strengthened among healthcare providers both at healthcare facilities and in the community, in order to intensify HIV retesting among eligible women until the end of breastfeeding period and to identify those who have no access to the healthcare system.

An effective communication strategy, involving community leaders, needs to be properly disseminated as a critical component of the global effort in HIV prevention and education of rural communities and women who do not have access to the conventional media. Additionally, PMTCT coverage estimates and programmatic targets should be revised considering the findings from population-based evidence.

Finally, in the era of sustainable development goals, all the efforts done so far will remain elusive unless multisectoral players, beyond the healthcare sector, work in synergy to address the identified socioeconomic factors, to empower women socially and economically, and to improve awareness regarding their HIV status and access the healthcare services for themselves and their children.

## Supporting information

S1 Appendix Children tested for HIV. (DOCX)

S1 Table Characteristics of the interviewed mothers disaggregated by infants/children with “unknown HIV exposure status,” “HIV-unexposed status,” and “HIV-exposed status”. (DOCX)

S2 Table Tables 1, 2 and 3 with all demographic, socioeconomic and health variables analysed. (DOCX)
