# Supplementary material for: High proportion of unknown HIV exposure status among children aged less than 2 years: An analytical study using the 2015 National AIDS Indicator Survey in Mozambique
Source: PLoS One. 2020 Apr 7;15(4):e0231143. doi: 10.1371/journal.pone.0231143 (PMC7138315; doi:10.1371/journal.pone.0231143)
Supplement: S1 Appendix — (DOCX) [file pone.0231143.s001.docx]

**S1 Appendix: Children tested for HIV**

In the 2015 IMASIDA Survey, mothers were asked if their infants/children <24 months of age were tested and received the HIV test result.

A total of 330 infants were tested for HIV and 294 received the test results, 1804 were not tested and 7 infants had missing information.

|  | | n | % |
| --- | --- | --- | --- |
| **Child tested for HIV** | No | 1804 | 84.5 |
|  | Yes | 330 | 15.5 |
|  | Missing | 7 |  |
|  | Total | 2141 | 100 |
| **Child received HIV test result** | No | 36 | 10.9 |
|  | Yes | 294 | 89.1 |
|  | Total | 330 | 100 |
|  | Not applicable | 1811 |  |
|  | Total | 2141 |  |
