# Supplementary material for: High proportion of unknown HIV exposure status among children aged less than 2 years: An analytical study using the 2015 National AIDS Indicator Survey in Mozambique
Source: PLoS One. 2020 Apr 7;15(4):e0231143. doi: 10.1371/journal.pone.0231143 (PMC7138315; doi:10.1371/journal.pone.0231143)
Supplement: S1 Table — (DOCX) [file pone.0231143.s002.docx]

**S2 Table: Characteristics of interviewed mothers disaggregated by infants/children with ‘Unknown HIV exposure status’, ‘HIV-unexposed status’, and ‘HIV-exposed status’**

|  |  | **All mothers**  **(n=2141)** | | | | **Unknown HIV exposure**  **(n=577)** | | | | **HIV-unexposed**  **(n=1435)** | | | | **HIV-exposed**  **(n=129)** | | | |
| --- | --- | --- | --- | --- | --- | --- | --- | --- | --- | --- | --- | --- | --- | --- | --- | --- | --- |
|  |  | n | % | 95% CI | | n | % | 95% CI | | n | % | 95% CI | | n | % | 95% CI | |
| Sex of household head | Male | 1348 | 63.0 | 60.9 | 65.1 | 395 | 68.5 | 66.5 | 70.5 | 892 | 62.2 | 60.1 | 64.3 | 61 | 47.3 | 45.2 | 49.4 |
|  | Female | 793 | 37.0 | 35.0 | 39.1 | 182 | 31.5 | 29.6 | 33.5 | 543 | 37.8 | 35.8 | 39.9 | 68 | 52.7 | 50.6 | 54.9 |
| Family members | ≤ 3 | 325 | 15.2 | 13.7 | 16.7 | 100 | 17.3 | 15.7 | 18.9 | 208 | 14.5 | 13.0 | 16.0 | 17 | 13.2 | 11.7 | 14.6 |
|  | 4+ | 1816 | 84.8 | 83.3 | 86.4 | 477 | 82.7 | 81.1 | 84.3 | 1227 | 85.5 | 84.0 | 87.1 | 112 | 86.8 | 85.4 | 88.3 |
| Mother's Age (years) | 15-19 | 406 | 19.0 | 17.3 | 20.6 | 116 | 20.1 | 18.4 | 21.8 | 281 | 19.6 | 17.9 | 21.3 | 9 | 7.0 | 5.9 | 8.1 |
|  | 20-24 | 607 | 28.4 | 26.4 | 30.3 | 152 | 26.3 | 24.5 | 28.2 | 434 | 30.2 | 28.3 | 32.2 | 21 | 16.3 | 14.7 | 17.9 |
|  | 25-29 | 448 | 20.9 | 19.2 | 22.7 | 117 | 20.3 | 18.6 | 22.0 | 299 | 20.8 | 19.1 | 22.6 | 32 | 24.8 | 23.0 | 26.7 |
|  | 30-34 | 313 | 14.6 | 13.1 | 16.1 | 72 | 12.5 | 11.1 | 13.9 | 206 | 14.4 | 12.9 | 15.9 | 35 | 27.1 | 25.2 | 29.0 |
|  | 35+ | 367 | 17.1 | 15.5 | 18.8 | 120 | 20.8 | 19.1 | 22.5 | 215 | 15.0 | 13.5 | 16.5 | 32 | 24.8 | 23.0 | 26.7 |
| Participation in family decisions | No | 1920 | 89.7 | 88.4 | 91.0 | 533 | 92.4 | 91.3 | 93.6 | 1273 | 88.7 | 87.4 | 90.1 | 114 | 88.4 | 87.0 | 89.8 |
|  | Yes | 221 | 10.3 | 9.0 | 11.6 | 44 | 7.6 | 6.5 | 8.8 | 162 | 11.3 | 9.9 | 12.6 | 15 | 11.6 | 10.3 | 13.0 |
| Mother's educational level | No education | 531 | 24.8 | 23.0 | 26.7 | 231 | 40.0 | 38.0 | 42.1 | 283 | 19.7 | 18.0 | 21.4 | 17 | 13.2 | 11.7 | 14.6 |
|  | Primary | 1135 | 53.0 | 50.9 | 55.2 | 311 | 53.9 | 51.8 | 56.1 | 745 | 51.9 | 49.8 | 54.1 | 79 | 61.2 | 59.2 | 63.4 |
|  | Secondary | 475 | 22.2 | 20.4 | 24.0 | 35 | 6.1 | 5.1 | 7.1 | 407 | 28.4 | 26.5 | 30.3 | 33 | 25.6 | 23.7 | 27.5 |
| Mother's job | No job | 1188 | 55.5 | 53.4 | 57.6 | 312 | 54.1 | 52.0 | 56.2 | 802 | 55.9 | 53.8 | 58.0 | 74 | 57.4 | 55.3 | 59.5 |
|  | With job | 953 | 44.5 | 42.4 | 46.7 | 265 | 45.9 | 43.8 | 48.1 | 633 | 44.1 | 42.0 | 46.3 | 55 | 42.6 | 40.5 | 44.8 |
| Mother's religion | Catholic | 502 | 23.5 | 21.7 | 25.3 | 150 | 26.1 | 24.2 | 28.0 | 329 | 22.9 | 21.2 | 24.7 | 23 | 17.8 | 16.2 | 19.5 |
|  | Islamic | 381 | 17.8 | 16.2 | 19.5 | 140 | 24.3 | 22.5 | 26.2 | 234 | 16.3 | 14.8 | 17.9 | 7 | 5.4 | 4.5 | 6.4 |
|  | Other Christian | 724 | 33.9 | 31.9 | 35.9 | 139 | 24.2 | 22.4 | 26.0 | 515 | 35.9 | 33.9 | 38.0 | 70 | 54.3 | 52.2 | 56.4 |
|  | Other | 531 | 24.8 | 23.0 | 26.7 | 146 | 25.4 | 23.5 | 27.3 | 356 | 24.8 | 23.0 | 26.7 | 29 | 22.5 | 20.7 | 24.3 |
|  | Missing | 3 |  |  |  | 2 |  |  |  | 1 |  |  |  | 0 |  |  |  |
| Mean of transportation | No | 1190 | 55.6 | 53.5 | 57.7 | 274 | 47.5 | 45.4 | 49.6 | 818 | 57.0 | 54.9 | 59.2 | 98 | 76.0 | 74.2 | 77.8 |
|  | Yes | 951 | 44.4 | 42.3 | 46.6 | 303 | 52.5 | 50.4 | 54.7 | 617 | 43.0 | 40.9 | 45.1 | 31 | 24.0 | 22.2 | 25.9 |
| Source of water at home | No piped water | 1325 | 61.9 | 59.8 | 64.0 | 463 | 80.2 | 78.6 | 82.0 | 815 | 56.8 | 54.7 | 58.9 | 47 | 36.4 | 34.4 | 38.5 |
|  | With piped water | 816 | 38.1 | 36.1 | 40.2 | 114 | 19.8 | 18.1 | 21.5 | 620 | 43.2 | 41.1 | 45.3 | 82 | 63.6 | 61.5 | 65.7 |
| Toilet | Not improved | 1863 | 87.0 | 85.6 | 88.5 | 555 | 96.2 | 95.4 | 97.1 | 1200 | 83.6 | 82.1 | 85.3 | 108 | 83.7 | 82.2 | 85.4 |
|  | Improved | 278 | 13.0 | 11.6 | 14.4 | 22 | 3.8 | 3.0 | 4.6 | 235 | 16.4 | 14.8 | 18.0 | 21 | 16.3 | 14.7 | 17.9 |
| Cooking fuel | Improved Cooking Fuel | 62 | 2.9 | 2.2 | 3.6 | 1 | .2 | 0.0 | 0.3 | 56 | 3.9 | 3.1 | 4.7 | 5 | 3.9 | 3.1 | 4.7 |
|  | Coal or Wood | 2053 | 95.9 | 95.0 | 96.8 | 571 | 99.0 | 98.5 | 99.5 | 1360 | 94.8 | 93.8 | 95.8 | 122 | 94.6 | 93.6 | 95.6 |
|  | Not applicable | 26 | 1.2 | 0.8 | 1.7 | 5 | .9 | 0.5 | 1.3 | 19 | 1.3 | 0.8 | 1.8 | 2 | 1.6 | 1.0 | 2.1 |
| Media utilization | No | 1476 | 68.9 | 67.0 | 71.0 | 483 | 83.7 | 82.1 | 85.3 | 916 | 63.8 | 61.8 | 65.9 | 77 | 59.7 | 57.6 | 61.8 |
|  | Yes | 665 | 31.1 | 29.1 | 33.0 | 94 | 16.3 | 14.7 | 17.9 | 519 | 36.2 | 34.1 | 38.2 | 52 | 40.3 | 38.2 | 42.4 |
| Mother travel in the past 12 moths | No | 1638 | 76.5 | 74.7 | 78.4 | 477 | 82.7 | 81.1 | 84.3 | 1084 | 75.5 | 73.7 | 77.4 | 77 | 59.7 | 57.6 | 61.8 |
|  | Yes | 503 | 23.5 | 21.7 | 25.3 | 100 | 17.3 | 15.7 | 18.9 | 351 | 24.5 | 22.6 | 26.3 | 52 | 40.3 | 38.2 | 42.4 |
| Region of residence | North | 628 | 29.3 | 27.4 | 31.3 | 240 | 41.6 | 39.5 | 43.7 | 378 | 26.3 | 24.5 | 28.2 | 10 | 7.8 | 6.6 | 8.9 |
|  | Center | 892 | 41.7 | 39.6 | 43.8 | 293 | 50.8 | 48.7 | 52.9 | 566 | 39.4 | 37.4 | 41.5 | 33 | 25.6 | 23.7 | 27.5 |
|  | South | 621 | 29.0 | 27.1 | 31.0 | 44 | 7.6 | 6.5 | 8.8 | 491 | 34.2 | 32.2 | 36.3 | 86 | 66.7 | 64.7 | 68.7 |
| Place of residence | Urban | 775 | 36.2 | 34.2 | 38.3 | 92 | 15.9 | 14.4 | 17.5 | 620 | 43.2 | 41.1 | 45.3 | 63 | 48.8 | 46.7 | 51.0 |
|  | Rural | 1366 | 63.8 | 61.8 | 65.9 | 485 | 84.1 | 82.5 | 85.7 | 815 | 56.8 | 54.7 | 58.9 | 66 | 51.2 | 49.0 | 53.3 |
| Time to reach health facility | Up to 30 minutes | 793 | 39.6 | 37.5 | 41.7 | 149 | 30.3 | 28.3 | 32.3 | 584 | 42.2 | 40.1 | 44.3 | 60 | 47.6 | 45.5 | 49.8 |
|  | >30 minutes | 1209 | 60.4 | 58.3 | 62.5 | 343 | 69.7 | 67.8 | 71.7 | 800 | 57.8 | 55.7 | 59.9 | 66 | 52.4 | 50.3 | 54.5 |
|  | Missing | 139 |  |  |  | 85 |  |  |  | 51 |  |  |  | 3 |  |  |  |
| Nr of live children | 1 | 526 | 24.6 | 22.7 | 26.4 | 154 | 26.7 | 24.8 | 28.6 | 356 | 24.8 | 23.0 | 26.7 | 16 | 12.4 | 11.0 | 13.8 |
|  | 2 | 397 | 18.5 | 16.9 | 20.2 | 80 | 13.9 | 12.4 | 15.3 | 301 | 21.0 | 19.3 | 22.7 | 16 | 12.4 | 11.0 | 13.8 |
|  | 3 | 338 | 15.8 | 14.2 | 17.3 | 83 | 14.4 | 12.9 | 15.9 | 223 | 15.5 | 14.0 | 17.1 | 32 | 24.8 | 23.0 | 26.7 |
|  | 4 | 261 | 12.2 | 10.8 | 13.6 | 61 | 10.6 | 9.3 | 11.9 | 179 | 12.5 | 11.1 | 13.9 | 21 | 16.3 | 14.7 | 17.9 |
|  | 5+ | 619 | 28.9 | 27.0 | 30.9 | 199 | 34.5 | 32.5 | 36.5 | 376 | 26.2 | 24.3 | 28.1 | 44 | 34.1 | 32.1 | 36.1 |
| ANC consultation (n) | None | 134 | 6.3 | 5.2 | 7.3 | 108 | 18.7 | 17.1 | 20.4 | 26 | 1.8 | 1.2 | 2.4 | 21 | 16.3 | 14.7 | 17.9 |
|  | 1-3 | 652 | 30.5 | 28.5 | 32.4 | 214 | 37.1 | 35.0 | 39.2 | 417 | 29.1 | 27.1 | 31.0 | 108 | 83.7 | 82.2 | 85.4 |
|  | 4+ | 1355 | 63.3 | 61.2 | 65.4 | 255 | 44.2 | 42.1 | 46.3 | 992 | 69.1 | 67.2 | 71.1 | 129 | 100.0 | 100.0 | 100.0 |
| Mother HIV test at ANC or Delivery | Not done | 346 | 16.2 | 14.6 | 17.7 | 272 | 47.1 | 45.0 | 49.3 | 46 | 3.2 | 2.5 | 4.0 | 28 | 21.7 | 20.0 | 23.5 |
|  | done | 1650 | 77.1 | 75.3 | 78.9 | 183 | 31.7 | 29.7 | 33.7 | 1366 | 95.2 | 94.3 | 96.2 | 101 | 78.3 | 76.5 | 80.1 |
|  | Unknown | 145 | 6.8 | 5.7 | 7.8 | 122 | 21.1 | 19.4 | 22.9 | 23 | 1.6 | 1.1 | 2.1 | 0 |  |  |  |
| Institutional Delivery | No | 550 | 25.7 | 23.8 | 27.6 | 256 | 44.4 | 42.3 | 46.5 | 276 | 19.2 | 17.6 | 20.9 | 18 | 14.0 | 12.5 | 15.4 |
|  | Yes | 1591 | 74.3 | 72.5 | 76.2 | 321 | 55.6 | 53.5 | 57.8 | 1159 | 80.8 | 79.1 | 82.5 | 111 | 86.0 | 84.6 | 87.6 |
| Place of delivery | Respondent's home | 472 | 22.1 | 20.3 | 23.9 | 227 | 39.6 | 37.5 | 41.7 | 231 | 16.1 | 14.6 | 17.7 | 14 | 10.9 | 9.5 | 12.2 |
|  | Other home | 43 | 2.0 | 1.4 | 2.6 | 20 | 3.5 | 2.7 | 4.3 | 23 | 1.6 | 1.1 | 2.1 | 0 |  |  |  |
|  | Hospital | 601 | 28.1 | 26.2 | 30.1 | 80 | 14.0 | 12.5 | 15.4 | 460 | 32.1 | 30.1 | 34.1 | 61 | 47.3 | 45.2 | 49.4 |
|  | Health center | 722 | 33.8 | 31.8 | 35.8 | 159 | 27.7 | 25.9 | 29.7 | 520 | 36.3 | 34.2 | 38.3 | 43 | 33.3 | 31.3 | 35.4 |
|  | Health post | 261 | 12.2 | 10.8 | 13.6 | 82 | 14.3 | 12.8 | 15.8 | 172 | 12.0 | 10.6 | 13.4 | 7 | 5.4 | 4.5 | 6.4 |
|  | Clinic | 7 | .3 | 0.1 | 0.6 | 0 |  |  |  | 7 | .5 | 0.2 | 0.8 | 0 |  |  |  |
|  | Other private | 6 | .3 | 0.1 | 0.5 | 1 | .2 | 0.0 | 0.4 | 4 | .3 | 0.1 | 0.5 | 1 | .8 | 0.4 | 1.1 |
|  | Other | 24 | 1.1 | 0.7 | 1.6 | 4 | .7 | 0.3 | 1.1 | 17 | 1.2 | 0.7 | 1.6 | 3 | 2.3 | 1.7 | 3.0 |
|  | Missing | 5 |  |  |  | 4 |  |  |  | 1 |  |  |  | 0 |  |  |  |
| Current age of last child | 0 | 1033 | 48.2 | 46.1 | 50.4 | 273 | 47.3 | 45.2 | 49.5 | 689 | 48.0 | 45.9 | 50.2 | 71 | 55.0 | 52.9 | 57.2 |
|  | 1 | 1014 | 47.4 | 45.2 | 49.5 | 289 | 50.1 | 48.0 | 52.2 | 673 | 46.9 | 44.8 | 49.1 | 52 | 40.3 | 38.2 | 42.4 |
|  | 2 | 94 | 4.4 | 3.5 | 5.3 | 15 | 2.6 | 1.9 | 3.3 | 73 | 5.1 | 4.2 | 6.0 | 6 | 4.7 | 3.8 | 5.5 |
| Sex of last child | Male | 1051 | 49.1 | 47.0 | 51.3 | 287 | 49.7 | 47.6 | 51.9 | 707 | 49.3 | 47.2 | 51.4 | 57 | 44.2 | 42.1 | 46.3 |
|  | Female | 1090 | 50.9 | 48.8 | 53.1 | 290 | 50.3 | 48.1 | 52.4 | 728 | 50.7 | 48.6 | 52.9 | 72 | 55.8 | 53.7 | 58.0 |
| Breastfeeding duration of last child | ≤ 6 months | 62 | 2.9 | 2.2 | 3.6 | 11 | 1.9 | 1.3 | 2.5 | 32 | 2.2 | 1.6 | 2.9 | 19 | 14.7 | 13.2 | 16.2 |
|  | ≤ 12 months | 125 | 5.9 | 4.9 | 6.9 | 38 | 6.6 | 5.6 | 7.7 | 75 | 5.3 | 4.3 | 6.2 | 12 | 9.3 | 8.1 | 10.5 |
|  | ≤ 18 months | 148 | 7.0 | 5.9 | 8.0 | 27 | 4.7 | 3.8 | 5.6 | 118 | 8.3 | 7.1 | 9.4 | 3 | 2.3 | 1.7 | 3.0 |
|  | Currently breastfeeding | 1769 | 83.1 | 81.5 | 84.8 | 494 | 86.4 | 84.9 | 87.9 | 1186 | 83.1 | 81.5 | 84.8 | 89 | 69.0 | 67.0 | 71.0 |
|  | Never breastfed | 24 | 1.1 | 0.7 | 1.6 | 2 | .3 | 0.1 | 0.6 | 16 | 1.1 | 0.7 | 1.6 | 6 | 4.7 | 3.8 | 5.5 |
|  | Missing | 13 |  |  |  | 5 |  |  |  | 8 |  |  |  | 0 |  |  |  |
| Infant's postnatal visit within 2 months of life | No | 421 | 19.7 | 18.0 | 21.4 | 189 | 33.0 | 31.0 | 35.0 | 221 | 15.4 | 13.9 | 17.0 | 11 | 8.6 | 7.4 | 9.8 |
|  | Yes | 1648 | 77.2 | 75.4 | 79.0 | 355 | 62.0 | 59.9 | 64.1 | 1177 | 82.1 | 80.5 | 83.8 | 116 | 90.6 | 89.4 | 91.9 |
|  | Don't know | 66 | 3.1 | 2.4 | 3.8 | 29 | 5.1 | 4.1 | 6.0 | 36 | 2.5 | 1.8 | 3.2 | 1 | .8 | 0.4 | 1.2 |
|  | Missing | 6 |  |  |  | 4 |  | 0.0 | 0.0 | 1 |  |  |  | 1 |  |  |  |
| Child Immunization up-to-date | No | 468 | 21.9 | 20.1 | 23.6 | 200 | 34.7 | 32.6 | 36.7 | 252 | 17.6 | 15.9 | 19.2 | 16 | 12.4 | 11.0 | 13.8 |
|  | Yes | 1673 | 78.1 | 76.4 | 80.0 | 377 | 65.3 | 63.3 | 67.4 | 1183 | 82.4 | 80.8 | 84.1 | 113 | 87.6 | 86.2 | 89.1 |
| Currently pregnant | No or unsure | 2075 | 96.9 | 96.2 | 97.7 | 552 | 95.7 | 94.8 | 96.6 | 1397 | 97.4 | 96.7 | 98.1 | 126 | 97.7 | 97.0 | 98.4 |
|  | Yes | 66 | 3.1 | 2.4 | 3.8 | 25 | 4.3 | 3.5 | 5.2 | 38 | 2.6 | 2.0 | 3.3 | 3 | 2.3 | 1.7 | 3.0 |
| Utilization of Health Services (ANC Delivery and PNC) | No | 1176 | 54.9 | 52.8 | 57.1 | 439 | 76.1 | 74.3 | 78.0 | 696 | 48.5 | 46.4 | 50.7 | 41 | 31.8 | 29.8 | 33.8 |
|  | Yes | 965 | 45.1 | 43.0 | 47.2 | 138 | 23.9 | 22.1 | 25.7 | 739 | 51.5 | 49.4 | 53.7 | 88 | 68.2 | 66.2 | 70.2 |
| Self-reported HIV status of previous HIV test | Positive | 141 | 6.6 | 5.5 | 7.7 | 0 | - | - | - | 12 | .8 | 0.5 | 1.2 | 129 | 100.0 | - | - |
|  | Negative | 1520 | 71 | 69.1 | 73.0 | 97 | 16.8 | 15.2 | 18.4 | 1423 | 99.2 | 98.8 | 99.6 | - | - | - | - |
|  | Indeterminate | 10 | 0.5 | 0.2 | 0.8 | 10 | 1.7 | 1.2 | 2.3 | - | - | - | - | - | - | - | - |
|  | Refused to answer | 7 | 0.3 | 0.1 | 0.5 | 7 | 1.2 | 0.7 | 1.7 | - | - | - | - | - | - | - | - |
|  | Did not receive result of last test | 81 | 3.8 | 3.0 | 4.6 | 81 | 14.0 | 12.6 | 15.5 | - | - | - | - | - | - | - | - |
|  | Never tested | 382 | 17.8 | 16.2 | 19.4 | 382 | 66.2 | 64.2 | 68.3 | - | - | - | - | - | - | - | - |
| Child exposed to HIV | Not exposed | 1435 | 67.0 | 65.0 | 69.1 | - | - | - | - | - | - | - | - | - | - | - | - |
|  | Exposed | 129 | 6.0 | 5.0 | 7.0 | - | - | - | - | - | - | - | - | - | - | - | - |
|  | Unknown | 577 | 27.0 | 25.1 | 28.9 | - | - | - | - | - | - | - | - | - | - | - | - |
|  | Total | 2141 | 100.0 |  |  | - | - | - | - | - | - | - | - | - | - | - | - |
