# Supplementary material for: High proportion of unknown HIV exposure status among children aged less than 2 years: An analytical study using the 2015 National AIDS Indicator Survey in Mozambique
Source: PLoS One. 2020 Apr 7;15(4):e0231143. doi: 10.1371/journal.pone.0231143 (PMC7138315; doi:10.1371/journal.pone.0231143)
Supplement: S2 Table — (DOCX) [file pone.0231143.s003.docx]

**S3 Tables: Tables 1, 2, 3 with all demographic, socioeconomic and health variables**

Full table 1- Characteristics of interviewed mothers- Mozambique 2015 (N= 2141);

Full table 2 Factors associated with child unknown HIV exposure status - Mozambique 2015

Full table 3 Factors associated with the condition ‘HIV exposed child’- Mozambique 2015

**Table 1.** **Characteristics of interviewed mothers- Mozambique 2015 (N= 2141)**

|  |  | **All (n=2141)** | | | |
| --- | --- | --- | --- | --- | --- |
|  |  | **n** | **%** | **95% CI** | |
| **Sex of household head** | Male | 1348 | 63.0 | 60.9 | 65.1 |
|  | Female | 793 | 37.0 | 35.0 | 39.1 |
| **Family members** | ≤ 3 | 325 | 15.2 | 13.7 | 16.7 |
|  | 4+ | 1816 | 84.8 | 83.3 | 86.4 |
| **Mother's Age (years)** | 15-19 | 406 | 19.0 | 17.3 | 20.6 |
|  | 20-24 | 607 | 28.4 | 26.4 | 30.3 |
|  | 25-29 | 448 | 20.9 | 19.2 | 22.7 |
|  | 30-34 | 313 | 14.6 | 13.1 | 16.1 |
|  | 35+ | 367 | 17.1 | 15.5 | 18.8 |
| **Participation in family decisions** | No | 1920 | 89.7 | 88.4 | 91.0 |
|  | Yes | 221 | 10.3 | 9.0 | 11.6 |
| **Mother's educational level** | No education | 531 | 24.8 | 23.0 | 26.7 |
|  | Primary | 1135 | 53.0 | 50.9 | 55.2 |
|  | Secondary | 475 | 22.2 | 20.4 | 24.0 |
| **Mother's job** | No job | 1188 | 55.5 | 53.4 | 57.6 |
|  | With job | 953 | 44.5 | 42.4 | 46.7 |
| **Mother's religion** | Catholic | 502 | 23.5 | 21.7 | 25.3 |
|  | Islamic | 381 | 17.8 | 16.2 | 19.5 |
|  | Other Christian | 724 | 33.9 | 31.9 | 35.9 |
|  | Other | 531 | 24.8 | 23.0 | 26.7 |
|  | Missing | 3 |  |  |  |
| **Mean of transportation** | No | 1190 | 55.6 | 53.5 | 57.7 |
|  | Yes | 951 | 44.4 | 42.3 | 46.6 |
| **Source of water at home** | No piped water | 1325 | 61.9 | 59.8 | 64.0 |
|  | With piped water | 816 | 38.1 | 36.1 | 40.2 |
| **Toilet** | Not improved | 1863 | 87.0 | 85.6 | 88.5 |
|  | Improved | 278 | 13.0 | 11.6 | 14.4 |
| **Cooking fuel** | Improved | 62 | 2.9 | 2.2 | 3.6 |
|  | Coal or Wood | 2053 | 95.9 | 95.0 | 96.8 |
|  | Not applicable | 26 | 1.2 | 0.8 | 1.7 |
| **Media utilization** | No | 1476 | 68.9 | 67.0 | 71.0 |
|  | Yes | 665 | 31.1 | 29.1 | 33.0 |
| **Mother travel in the past 12 moths** | No | 1638 | 76.5 | 74.7 | 78.4 |
|  | Yes | 503 | 23.5 | 21.7 | 25.3 |
| **Region of residence** | North | 628 | 29.3 | 27.4 | 31.3 |
|  | Center | 892 | 41.7 | 39.6 | 43.8 |
|  | South | 621 | 29.0 | 27.1 | 31.0 |
| **Place of residence** | Urban | 775 | 36.2 | 34.2 | 38.3 |
|  | Rural | 1366 | 63.8 | 61.8 | 65.9 |
| **Time to reach health facility** | Up to 30 minutes | 793 | 39.6 | 37.5 | 41.7 |
|  | >30 minutes | 1209 | 60.4 | 58.3 | 62.5 |
|  | Missing | 139 |  |  |  |
| **Nr of live children** | 1 | 526 | 24.6 | 22.7 | 26.4 |
|  | 2 | 397 | 18.5 | 16.9 | 20.2 |
|  | 3 | 338 | 15.8 | 14.2 | 17.3 |
|  | 4 | 261 | 12.2 | 10.8 | 13.6 |
|  | 5+ | 619 | 28.9 | 27.0 | 30.9 |
| **ANC consultation (n)** | None | 134 | 6.3 | 5.2 | 7.3 |
|  | 1-3 | 652 | 30.5 | 28.5 | 32.4 |
|  | 4+ | 1355 | 63.3 | 61.2 | 65.4 |
| **Mother HIV test at ANC or Delivery** | Not done | 346 | 16.2 | 14.6 | 17.7 |
|  | done | 1650 | 77.1 | 75.3 | 78.9 |
|  | Unknown | 145 | 6.8 | 5.7 | 7.8 |
| **Institutional Delivery** | No | 550 | 25.7 | 23.8 | 27.6 |
|  | Yes | 1591 | 74.3 | 72.5 | 76.2 |
| **Place of delivery** | Respondent's home | 472 | 22.1 | 20.3 | 23.9 |
|  | Other home | 43 | 2.0 | 1.4 | 2.6 |
|  | Hospital | 601 | 28.1 | 26.2 | 30.1 |
|  | Health center | 722 | 33.8 | 31.8 | 35.8 |
|  | Health post | 261 | 12.2 | 10.8 | 13.6 |
|  | Clinic | 7 | .3 | 0.1 | 0.6 |
|  | Other private | 6 | .3 | 0.1 | 0.5 |
|  | Other | 24 | 1.1 | 0.7 | 1.6 |
|  | Missing | 5 |  |  |  |
| **Current age of last child** | 0 | 1033 | 48.2 | 46.1 | 50.4 |
|  | 1 | 1014 | 47.4 | 45.2 | 49.5 |
|  | 2 | 94 | 4.4 | 3.5 | 5.3 |
| **Sex of last child** | Male | 1051 | 49.1 | 47.0 | 51.3 |
|  | Female | 1090 | 50.9 | 48.8 | 53.1 |
| **Breastfeeding duration of last child** | ≤ 6 months | 62 | 2.9 | 2.2 | 3.6 |
|  | ≤ 12 months | 125 | 5.9 | 4.9 | 6.9 |
|  | ≤ 18 months | 148 | 7.0 | 5.9 | 8.0 |
|  | Currently breastfeeding | 1769 | 83.1 | 81.5 | 84.8 |
|  | Never breastfed | 24 | 1.1 | 0.7 | 1.6 |
|  | Missing | 13 |  |  |  |
| **Infant's postnatal visit within 2 months of life** | No | 421 | 19.7 | 18.0 | 21.4 |
|  | Yes | 1648 | 77.2 | 75.4 | 79.0 |
|  | Don't know | 66 | 3.1 | 2.4 | 3.8 |
|  | Missing | 6 |  |  |  |
| **Child Immunization up-to-date** | No | 468 | 21.9 | 20.1 | 23.6 |
|  | Yes | 1673 | 78.1 | 76.4 | 80.0 |
| **Currently pregnant** | No or unsure | 2075 | 96.9 | 96.2 | 97.7 |
|  | Yes | 66 | 3.1 | 2.4 | 3.8 |
| **Utilization of Health Services (ANC, Maternity and PNC)** | No | 1176 | 54.9 | 52.8 | 57.1 |
|  | Yes | 965 | 45.1 | 43.0 | 47.2 |
| **Self-reported HIV status of previous HIV test** | Positive | 141 | 6.6 | 5.5 | 7.7 |
|  | Negative | 1520 | 71 | 69.1 | 73.0 |
|  | Indeterminate | 10 | 0.5 | 0.2 | 0.8 |
|  | Refused to answer | 7 | 0.3 | 0.1 | 0.5 |
|  | Did not receive result | 81 | 3.8 | 3.0 | 4.6 |
|  | Never tested | 382 | 17.8 | 16.2 | 19.4 |
| **Child exposed to HIV** | Not exposed | 1435 | 67.0 | 65.0 | 69.1 |
|  | Exposed | 129 | 6.0 | 5.0 | 7.0 |
|  | Unknown | 577 | 27.0 | 25.1 | 28.9 |
|  | Total | 2141 | 100.0 |  |  |

ANC: Antenatal care; PNC: Postnatal care

**Table 2. Factors associated with child unknown HIV exposure status - Mozambique 2015**

| **Unknown HIV exposure status in child** | | **Bivariate analysis** | | | | **Multivariate analysis** | | | |
| --- | --- | --- | --- | --- | --- | --- | --- | --- | --- |
|  | | ***p value*** | **OR** | **95% CI** | | ***P value*** | **Adjusted OR** | **95% CI** | |
|  |  |  |  | **Lower** | **Upper** |  |  | **Lower** | **Upper** |
| **Sex of household head** | Male vs. Female | .027 | 1.317 | 1.032 | 1.680 | .104 | 1.285 | .950 | 1.738 |
| **Family members (nr)*** | ≤3 vs. 4+ people | .235 | 1.211 | .882 | 1.663 | - | - | - | - |
| **Participation in family decision*** | No vs. Yes | .052 | 1.547 | .996 | 2.403 | - | - | - | - |
| **Mother's job*** | No vs. Yes | .674 | .934 | .679 | 1.285 | - | - | - | - |
| **Mother's age (years)** | 15-19 vs. 35+ | .155 | .773 | .543 | 1.102 | .318 | .731 | .394 | 1.354 |
|  | 20-24 vs. 35+ | .097 | .744 | .524 | 1.056 | .827 | .944 | .563 | 1.582 |
|  | 25-29 vs. 35+ | .076 | .724 | .507 | 1.035 | .552 | .873 | .558 | 1.368 |
|  | 30-34 vs. 35+ | .005 | .560 | .372 | .841 | .212 | .736 | .454 | 1.192 |
| **Highest educational level** | No education vs. Secondary | .000 | 7.986 | 4.674 | 13.643 | .004 | 2.726 | 1.386 | 5.361 |
|  | Primary vs. Secondary | .000 | 4.475 | 2.812 | 7.120 | .031 | 1.826 | 1.057 | 3.153 |
| **Religion** | Catholic vs. Other | .274 | 1.212 | .858 | 1.713 | .518 | .851 | .521 | 1.389 |
|  | Islamic vs. Other | .162 | 1.361 | .883 | 2.096 | .822 | 1.077 | .564 | 2.056 |
|  | Other Christian vs. Other | .022 | .667 | .471 | .944 | .243 | .807 | .562 | 1.158 |
| **Wealth index** | Poorest vs. Richest | .000 | 11.195 | 6.380 | 19.643 | .391 | 1.464 | .611 | 3.505 |
|  | Poorer vs. Richest | .000 | 11.234 | 6.313 | 19.993 | .117 | 1.985 | .842 | 4.678 |
|  | Middle vs. Richest | .000 | 8.556 | 4.703 | 15.564 | .168 | 1.787 | .781 | 4.087 |
|  | Richer vs. Richest | .018 | 2.178 | 1.146 | 4.138 | .876 | 1.059 | .511 | 2.198 |
| **Place of residence** | Urban vs. Rural | .000 | .205 | .144 | .292 | .004 | .419 | .233 | .753 |
| **Region of residence** | North vs. South | .000 | 8.408 | 5.381 | 13.138 | .000 | 4.411 | 2.181 | 8.919 |
|  | Center vs. South | .000 | 6.959 | 4.573 | 10.588 | .000 | 3.015 | 1.861 | 4.886 |
| **Number of live children** | 1 vs. 5+ | .587 | .916 | .668 | 1.257 | .044 | 1.810 | 1.016 | 3.225 |
|  | 2 vs. 5+ | .016 | .641 | .447 | .921 | .907 | .968 | .559 | 1.676 |
|  | 3 vs. 5+ | .437 | .838 | .535 | 1.311 | .302 | 1.254 | .815 | 1.928 |
|  | 4 vs. 5+ | .016 | .599 | .395 | .909 | .047 | .606 | .370 | .993 |
| **Current age of last child (yr)** | 0 vs. 2 | .040 | 1.859 | 1.029 | 3.358 | .086 | 1.926 | .911 | 4.075 |
|  | 1 vs. 2 | .017 | 2.084 | 1.139 | 3.811 | .039 | 2.107 | 1.039 | 4.276 |
| **Breastfeeding duration of last child** | ≤ 6 months vs. Never | .090 | 3.633 | .817 | 16.157 | .067 | 3.633 | .911 | 14.488 |
|  | ≤ 12 months vs. Never | .019 | 5.333 | 1.315 | 21.630 | .016 | 4.486 | 1.321 | 15.234 |
|  | ≤ 18 months vs. Never | .147 | 2.873 | .688 | 12.001 | .239 | 2.286 | .576 | 9.074 |
|  | Still breastfeeding vs. Never | .024 | 4.671 | 1.231 | 17.718 | .160 | 2.296 | .718 | 7.341 |
| **Time to reach health facility** | Up to 30 minutes vs. ≥ 30 minutes | .011 | .648 | .465 | .904 | .365 | 1.172 | .831 | 1.653 |
| **ANC consultation (n)^#^** | 1-3 vs. 4+ | .000 | 2.165 | 1.606 | 2.918 | - | - | - | - |
| **Baby post-natal visit within 2 months of life*** | No vs. Don't know | .814 | 1.080 | .568 | 2.053 | - | - | - | - |
|  | Yes vs. Don't know | .254 | .701 | .381 | 1.293 | - | - | - | - |
| **Utilization of health services (ANC, Maternity, PNC)** | No vs. Yes | .000 | 2.640 | 1.852 | 3.763 | .000 | 1.905 | 1.422 | 2.552 |
| **Child Immunization up-to-date** | No vs. Yes | .000 | 2.162 | 1.595 | 2.930 | .161 | 1.246 | .916 | 1.695 |
| **Travel in the last 12 months** | No vs. Yes | .053 | 1.369 | .995 | 1.884 | - | - | - | - |

*Variable not analyzed in the Multivariate analysis because its association was not significative in the bivariate analysis.

^#^ Variable not analyzed in the Multivariate analysis because it is part of the variable “Utilization Health Service” which was analyzed in the Multivariate analysis.

ANC: Antenatal care; PNC: Postnatal care

**Table 3. Factors associated with the condition ‘HIV exposed child’- Mozambique 2015**

| **HIV exposed child** | | **Bivariate analysis** | | | | **Multivariate analysis** | | | | |
| --- | --- | --- | --- | --- | --- | --- | --- | --- | --- | --- |
|  | | ***p value*** | **OR** | **95% CI** | | ***P value*** | **Adjusted**  **OR** | **95% CI** | | |
|  |  |  |  | **Lower** | **Upper** |  |  | **Lower** | | **Upper** |
| **Sex of household head** | Male vs. Female | .000 | .472 | .315 | .707 | .000 | .418 | .261 | | .667 |
| **Family members (nr)*** | ≤3 people vs. 4+ | .727 | .899 | .495 | 1.634 | - | - | - | | - |
| **Mother's age (years)** | 15-19 vs. 35+ | .003 | .277 | .120 | .642 | .208 | .393 | .091 | | 1.687 |
|  | 20-24 vs. 35+ | .003 | .334 | .163 | .684 | .055 | .416 | .170 | | 1.018 |
|  | 25-29 vs. 35+ | .259 | .666 | .328 | 1.352 | .412 | .720 | .327 | | 1.584 |
|  | 30-34 vs. 35+ | .848 | 1.063 | .567 | 1.995 | .655 | 1.169 | .587 | | 2.327 |
| **Number of live children** | 1 vs. 5+ | .002 | .393 | .216 | .715 | .316 | .553 | .173 | | 1.764 |
|  | 2 vs. 5+ | .069 | .532 | .270 | 1.050 | .368 | .677 | .289 | | 1.585 |
|  | 3 vs. 5+ | .697 | 1.120 | .633 | 1.982 | .612 | 1.205 | .584 | | 2.484 |
|  | 4 vs. 5+ | .757 | 1.099 | .601 | 2.011 | .247 | 1.492 | .756 | | 2.943 |
| **Highest educational level*** | No education vs. Secondary | .055 | .518 | .265 | 1.015 | - | - | - | | - |
|  | Primary vs. Secondary | .869 | 1.045 | .621 | 1.759 | - | - | - | | - |
| **Source of water at home**** | No piped water vs. Piped water | .000 | .413 | .259 | .658 | - | - | - | | - |
| **Toilet type**** | not improved vs. Improved | .313 | .718 | .377 | 1.368 | - | - | - | | - |
| **Media utilization**** | No vs. Yes | .147 | .726 | .471 | 1.119 | - | - | - | | - |
| **Cooking fuel**** | Improved vs. Not applicable | .549 | .570 | .090 | 3.599 | - | - | - | | - |
|  | Coal or Wood vs. Not applicable | .660 | .710 | .153 | 3.289 | - | - | - | | - |
| **Participation in family decision*** | No vs. Yes | .970 | 1.013 | .527 | 1.945 | - | - | - | | - |
| **Mother job*** | No job vs. Yes job | .992 | .998 | .640 | 1.555 | - | - | - | | - |
| **Wealth index** | Poorest vs. Richest | .001 | .163 | .055 | .481 | .161 | .357 | .084 | | 1.512 |
|  | Poorer vs. Richest | .000 | .134 | .044 | .406 | .056 | .296 | .085 | | 1.032 |
|  | Middle vs. Richest | .533 | .820 | .438 | 1.534 | .116 | 1.870 | .856 | | 4.083 |
|  | Richer vs. Richest | .273 | .754 | .454 | 1.252 | .814 | .932 | .516 | | 1.682 |
| **Place of residence** | Urban vs. Rural | .049 | 1.553 | 1.002 | 2.405 | .750 | .910 | .509 | | 1.628 |
| **Region of residence** | North vs. South | .000 | .135 | .058 | .314 | .028 | .264 | .080 | | .867 |
|  | Center vs. South | .000 | .304 | .184 | .503 | .006 | .430 | .235 | | .787 |
| **Religion** | Catholic vs. 4 Other | .640 | .849 | .425 | 1.693 | .280 | 1.513 | .712 | | 3.214 |
|  | Islamic vs. 4 Other | .046 | .351 | .126 | .980 | .654 | .748 | .209 | | 2.679 |
|  | Other Christian vs. Other | .071 | 1.619 | .959 | 2.735 | .163 | 1.515 | .845 | | 2.715 |
| **Current age of last child (yr)** | 0 vs. 2 | .562 | 1.320 | .516 | 3.377 | - | - | - | | - |
|  | 1 vs. 2 | .964 | .978 | .370 | 2.586 | - | - | - | | - |
| **Breastfeeding duration of last child** | ≤ 6 months vs. Never | .191 | 2.352 | .651 | 8.492 | .105 | 3.459 | .771 | | 15.515 |
|  | ≤ 12 months vs. Never | .195 | .453 | .136 | 1.504 | .630 | .698 | .161 | | 3.027 |
|  | ≤ 18 months vs. Never | .004 | .081 | .015 | .436 | .009 | .070 | .010 | | .512 |
|  | Still breastfeeding vs. Never | .004 | .200 | .068 | .587 | .101 | .342 | .095 | | 1.234 |
| **Time to reach health facility** | Up to 30 minutes vs. ≥ 30 minutes | .029 | 1.652 | 1.052 | 2.595 | .544 | 1.160 | .718 | | 1.875 |
| **Utilization Health Service (ANC, Maternity, PNC)** | No vs. Yes | .001 | .436 | .265 | .718 | .007 | .520 | .323 | | .836 |
| **Child Immunization up-to-date*** | No vs. Yes | .188 | .648 | .340 | 1.237 | - | - | - | | - |
| **Currently pregnant*** | No or unsure vs. Yes | .632 | 1.343 | .400 | 4.516 | - | - | - | | - |
| **ANC consultation (n)#** | 1-3 vs. 4+ | .003 | .414 | .234 | .735 | - | - | - | | - |
| **Institutional delivery*** | No vs. Yes | .064 | .575 | .319 | 1.033 | - | - | - | | - |
| **Baby post-natal visit within 2 months of life*** | No vs. Don't know | .623 | 1.639 | .227 | 11.836 | - | - | - | | - |
|  | Yes vs. Don't know | .455 | 2.051 | .309 | 13.600 | - | - | - | | - |
| **Travelled in the last 12m** | No vs. Yes | .000 | .398 | .264 | .601 | .282 | .769 | .475 | | 1.244 |
| *Variable not analyzed in the Multivariate analysis because it was not significative in the bivariate | | | | | | | | |  |  |
| **In the multivariate analysis the variable was combined in the ‘wealth index’ variable  # In the multivariate analysis the variable is part of variable Utilization Health Service. | | | | | | |  | |  |  |

ANC: Antenatal care; PNC: Postnatal care
